# Sequence variability of Rhizobiales orthologs and relationship with physico-chemical characteristics of proteins

**DOI:** 10.1186/1745-6150-6-48

**Published:** 2011-10-04

**Authors:** Humberto Peralta, Gabriela Guerrero, Alejandro Aguilar, Jaime Mora

**Affiliations:** 1 Programa de Genómica Funcional de Procariotes, Centro de Ciencias Genómicas, Universidad Nacional Autónoma de México. Apdo. postal 565-A, Cuernavaca, Morelos, México

**Keywords:** rhizobia, comparative genomics, evolutionary rates, nonsynonymous substitution, adaptation

## Abstract

**Background:**

Chromosomal orthologs can reveal the shared ancestral gene set and their evolutionary trends. Additionally, physico-chemical properties of encoded proteins could provide information about functional adaptation and ecological niche requirements.

**Results:**

We analyzed 7080 genes (five groups of 1416 orthologs each) from Rhizobiales species (*S. meliloti, R. etli*, and *M. loti*, plant symbionts; *A. tumefaciens*, a plant pathogen; and *B. melitensis*, an animal pathogen). We evaluated their phylogenetic relationships and observed three main topologies. The first, with closer association of *R. etli *to *A. tumefaciens*; the second with *R. etli *closer to *S. meliloti*; and the third with *A. tumefaciens *and *S. meliloti *as the closest pair. This was not unusual, given the close relatedness of these three species. We calculated the synonymous (dS) and nonsynonymous (dN) substitution rates of these orthologs, and found that informational and metabolic functions showed relatively low dN rates; in contrast, genes from hypothetical functions and cellular processes showed high dN rates. An alternative measure of sequence variability, percentage of changes by species, was used to evaluate the most specific proportion of amino acid residues from alignments. When dN was compared with that measure a high correlation was obtained, revealing that much of evolutive information was extracted with the percentage of changes by species at the amino acid level. By analyzing the sequence variability of orthologs with a set of five properties (polarity, electrostatic charge, formation of secondary structures, molecular volume, and amino acid composition), we found that physico-chemical characteristics of proteins correlated with specific functional roles, and association of species did not follow their typical phylogeny, probably reflecting more adaptation to their life styles and niche preferences. In addition, orthologs with low dN rates had residues with more positive values of polarity, volume and electrostatic charge.

**Conclusions:**

These findings revealed that even when orthologs perform the same function in each genomic background, their sequences reveal important evolutionary tendencies and differences related to adaptation.

This article was reviewed by: Dr. Purificación López-García, Prof. Jeffrey Townsend (nominated by Dr. J. Peter Gogarten), and Ms. Olga Kamneva.

## Background

Orthologs are genes related to a common ancestor and derived from speciation events, and their identification is often based on the most significant bidirectional similarity [1]. However, Huynen and Bork [2] proposed that chromosomal gene order conservation (gene neighbor conservation, or synteny) is also a key factor in the identification of these genes. In another study, the determination of synteny was useful to enhance the accuracy of ortholog identification in mouse and human genomes [3]. Also, we reported an analysis of chromosomal orthologs in four species of Rhizobiales, and found that syntenic genes showed more sequence conservation, higher operon organization and network formation, and encoded key metabolic proteins as compared to genes without synteny [4]. We consider that these genes can be viewed as a model group to study the evolutionary differences and to assess the meaning of sequence variability of orthologs.

From the dogma that genes evolve and accumulate mostly neutral mutations [5], a question arises: how to detect and evaluate if specific residues in the protein sequences encoded by orthologs are responsible for adaptation? Gene evolution can be assessed by measuring single nucleotide mutations that change (nonsynonymous, dN) or do not change (synonymous, dS) the encoded amino acid. Genes with higher nonsynonymous to synonymous ratios (dN/dS > 1) are considered to have experienced positive selection, revealing an adaptive process at the molecular level [6,7]. In bacteria, however the majority of genes evolve under purifying selection (dN/dS < 1), which is a trend that eliminates deleterious mutations. Methods in the literature to measure dN and dS rates make several inferences and use probabilistic models [7,8]. Evolutionary studies commonly stop there, but it is important to further study the significance of ortholog variability, considered as neutral, and trying to obtain information about how the physico-chemical properties of proteins are defined by specific sequences. We proposed that important evolutive information can be extracted directly from protein sequences through detection of the most specific residue in a given position for each species in multiple alignments of orthologs, which we represent as the percentage of change by species, originally termed as the species signature [4]. We used that measure in conjunction with the series of values reported by Atchley and collaborators [9], that consist of sets of numeric values for amino acid variability, organized by physico-chemical property (namely polarity, secondary structure, molecular volume, amino acid composition, and electrostatic charge), which summarize data from 54 amino acid attributes.

The Rhizobiales is a versatile alpha-proteobacterial Order, with members showing diversity in genomic structure, metabolic processes, and lifestyle [10-12]. The Rhizobiales include plant facultative (*Agrobacterium*) and animal obligate pathogens (*Brucella*, *Bartonella*), facultative symbionts (*Sinorhizobium, Mesorhizobium, Rhizobium*, and *Bradyrhizobium*) and free-living organisms (*Rhodopseudomonas palustris *and the plant rhizobia) [13-15]. Several complete genome sequences of Rhizobiales are available, including *S. meliloti *[16], *M. loti *[17], *A. tumefaciens *[10,12], *R. etli *[18], and *B. melitensis *[11]. Despite their described versatility, the chromosomal orthologs in these species have enough conservation of the characteristics that define the species derived from the common ancestor in the alpha-proteobacterial Order [4].

As a continuation of our previous work [4], here we analyze the chromosomal orthologs of five Rhizobiales (*S. meliloti, R. etli, A. tumefaciens, B. melitensis *and *M. loti*) to evaluate the sequence variability among orthologs and the relationship with phylogeny and physico-chemical characteristics of proteins. In this genomic study, we used the series of values of physico-chemical properties reported by Atchley *et al. *[9], to define how the specific properties of proteins vary according to differences in amino acid residues, and also to determine the significance of the percentage of changes by species, in relation to variability. The aim of this study were to analyze the evolution of common orthologs in rhizobiales species, to extract the specific changes in their sequences, in relation to other evolutionary measures and to assess, theoretically, the physico-chemical impact of these changes. We hypothesize that many changes in sequences are not completely neutral, but define physico-chemical differences with a role in adaptation to species niches.

## Methods

### Detection of orthologs

The following genome sequences of Rhizobiales species were analyzed in this work: *Sinorhizobium meliloti *1021 (hereafter referred as *Sm *or *S*, Genbank accession number NC_003047), *Rhizobium etli *CFN42 (*Re *or *R*, NC_007761), *R. etli *strain CIAT652 (CIAT, NC_010994), *Agrobacterium tumefaciens *C58 (*At *or *A*; Cereon, NC_003062), *Mesorhizobium loti *MAFF303099 (*Ml *or *M*, NC_002678) and *Brucella melitensis *16M (*Bm *or *B*, NC_003317). These sequences were obtained from the NCBI web site (http://www.ncbi.nlm.nih.gov). Orthologs were detected by the best bidirectional hit between pairs of species, with *S. meliloti *as a reference, using the program Fasta34 [19] with the following parameters: an identity of at least 50%, overlapping by at least 150 nt, and an (E) score < 10^-5^. Orthologs in the five chromosomes were determined by combining the results of each pairwise comparison in order to get the common set. The correct ortholog correspondence among pair comparisons was manually checked. Additionally, to get a more strict ortholog assignation, the genes must have a synteny relationship (or conserved neighborhood), and for this we applied a method reported previously [4].

### Functional classification of the chromosomal orthologs

The functional annotation of the orthologs was that from *A. tumefaciens *C58-U. Washington annotation [10]; functions were manually checked against the other genomic functional annotations. The functions were classified into groups as follows. Metabolism: amino acid, nucleotide, fatty acid, and cofactor synthesis; central intermediary metabolism, and energy obtention; informational: DNA metabolism (including recombination, replication and repair), transcription, translation, and regulatory functions; processes: transport, cell envelope synthesis, and cellular processes (such as cell division, secretion and chemotaxis); and hypothetical and unknown functions.

### Identity, similarity and the percentage of changes by species (%chSp)

Similarity (pair comparisons) and common identity (multiple comparisons) were calculated with ClustalW alignments [20]. The percentage of changes by species (or %chSp), originally the species signature, was calculated as reported previously [4], and consists, briefly, in the amount of most specific residues for each ortholog, reported as a percentage, extracted from homologous positions in ClustalW multiple alignments. The cases when a position was considered part of the %chSp were: four identical residues and one different, three identical residues and two different (%chSp accounted for two species), two identical residues and three different (%chSp accounted for the three species), and five different residues (%chSp accounted for the five species). The percentage was calculated dividing by a common length. Gaps (internal differences) and non-aligned sequences (in the extremes of the sequences), were not considered and were trimmed with an *ad hoc *perl script, following accepted criteria.

### Phylogenetic analysis

Phylogenetic trees were obtained with the neighbor joining (NJ) and maximum likelihood (ML) methods. Sequences were first aligned with the ClustalW program, and the non-aligned segments and gaps were cut with *ad hoc *perl scripts. Unrooted phylogenies were calculated for each of the orthologs separately. For ML, we used the *proml *program, and for the NJ method, the *neighbor *program, both from the Phylip package v3.6 [21] by Joe Felsenstein, available on the web (http://www.evolution.genetics.washington.edu/phylip.html). The JTT model of substitution was applied. The ML and NJ methods gave similar results, and we chose to work with those obtained with ML.

### Synonymous and nonsynonymous substitution rates

Estimation of the number of synonymous (dS) and nonsynonymous (dN) substitutions per site was performed by ML analysis with the codeML program of the PAML package [22], used with model 1 (free ratio), runmode 0 (user tree), fixed branch length initial, and with the empirical F3 × 4 nucleotide frequencies model. The amino acid sequence alignments were traced back to their corresponding nucleotide sequences and the nonhomologous segments were trimmed with an *ad hoc *perl script. dN and dS values corresponded to the final branch. Genes with dN values above 0.4, or dS values above 5, were eliminated from the analysis. The orthologs were assigned to the group with low dN value if, according to the order of the five dN values of each ortholog, the second lowest value did not surpass the 0.05 dN limit. Otherwise, the ortholog was assigned to the high dN group. Codon usage tables were obtained from the Kazusa website (http://www.kazusa.or.jp/codon).

### Physico-chemical series of values for amino acids and prediction of transmembranal proteins

We used the series of values for amino acids (polarity, secondary structure, molecular volume, amino acid composition, and electrostatic charge) reported by Atchley *et al. *[9]. Amino acid values in the series of physico-chemical properties are positive or negative. To calculate the value for each sequence, the values for each residue were added, with sign conservation. Negative or positive values were obtained, with the exception of the amino acid composition, which always yields positive values. Clustering of results was performed with Multiple Experiment Viewer MeV TM4 [23], used with the normalized data from the values of physico-chemical properties, in hierarchical mode, with the Pearson rank correlation or Euclidean distance, and average linkage. To normalize values, all values in each row of data were multiplied by a scale factor S, so that the sum of the squares of the values in each row was 1 (a separate S factor is computed for each row). To normalize array (columns), a similar computation was performed. For the relationship of the %chSp with physico-chemical properties, the method described by Olivares-Hernandez *et al. *was followed [24]; briefly, range values of all properties and average of the %chSp of all species were calculated. To obtain clusters of products with high correlation values, the range of each property was divided by the average %chSp from the five organisms, the result was multiplied by log2 and clustered with the Multiple Experiment Viewer MeV TM4 program [23], using k-means and Euclidean distance. The TMAP program, version 46 [25], available in the EMBOSS package (http://www.emboss.org), was used to predict transmembranal segments in the protein sequences.

For functional analysis, orthologs were classified as positive or negative if values for each ortholog product from the five species had the same sign (in the case of amino acid composition, values were divided by half and assigned to low or high level groups). The resulting groups were: polarity, negative 725 sequences, positive 297; secondary structure, negative (representing abundance of alpha-helices) 530, positive (abundance of coils) 314; molecular volume, negative (representing products with small residues) 886, positive (products with bulky residues) 72; amino acid composition, low (represents atypical composition) 612, high (very typical composition) 621; electrostatic charge, negative 613, positive 191.

### Statistical analysis

The Mann-Whitney test was used to evaluate differences in frequency distributions and was performed with Minitab v12 (Minitab, Inc.). The Fisher's exact tests for significant differences in proportions were calculated with Oyvind Langsrud's page (http://www.langsrud.com/fisher.htm). Pearson correlation coefficients were calculated with Minitab v12.

## Results

We attempted to use the analysis of ortholog sequence variability by the percentage of changes by species (%chSp) in Rhizobiales genomes [4] as an instrument to determine genomic differences among groups of species, and evolutive coherence. This is connected with the phylogenetic relationships of the species, but also with the chemical nature of amino acid substitutions in proteins. Thus, we assessed the relationship of sequence variability with phylogeny and physico-chemical properties of a set of 1416 orthologs from five species of Rhizobiales (*S. meliloti, R. etli, A. tumefaciens, B. melitensis *and *M. loti*). We applied a measure we proposed, termed the percentage of changes by species, originally species signature (defined as the normalized number of residues in the ortholog sequence that occur only once in individual alignment columns) [4], intended to extract species-specific information, possibly related to the adaptation to niche, and compared the %chSp with the evolutionary rates of the orthologs (synonymous and nonsynonymous substitution rates). Additionally, we used five series of values for amino acid properties (polarity, secondary structure formation, volume, amino acid composition and electrostatic charge) reported by Atchley *et al. *[9], to explore the relationships of physico-chemical properties of residues encoded in the orthologs with phylogeny, evolutionary rates and function.

### The percentage of changes by species (%chSp) and relationships with function and dN and dS

**Figure 1:**
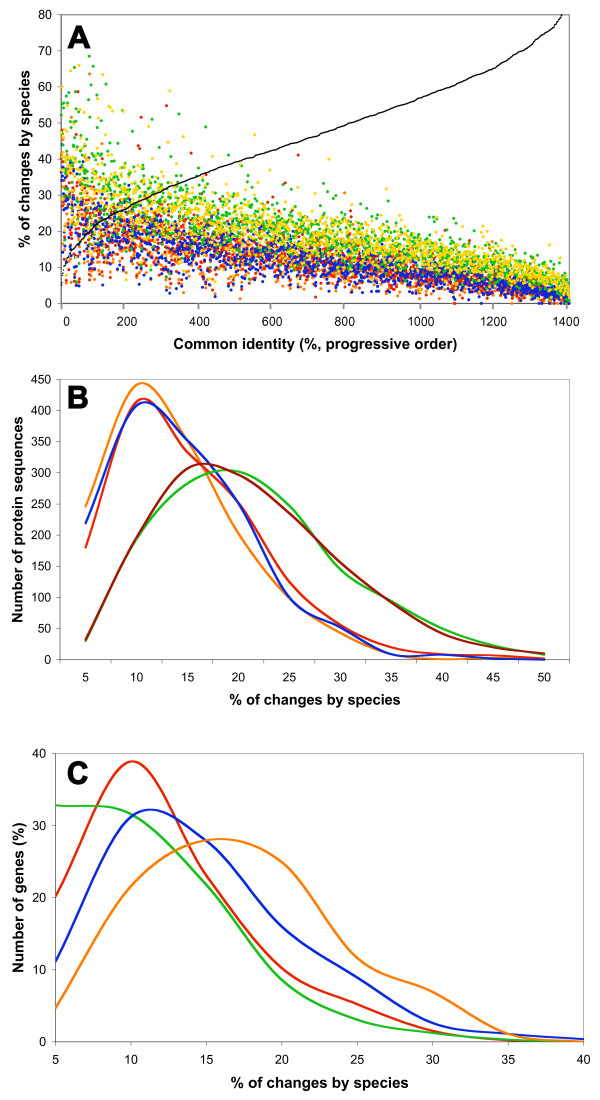
**The percentage of changes by species (%chSp) of 1416 chromosomal orthologs from five Rhizobiales**. The panels show 1416 orthologs for each of the species (7080 ortholog products in total). A, the %chSp arranged progressively according to the % of common identity of each ortholog from the five organisms (black line). Color dots: orange, *R. etli*; red, *A. tumefaciens*; blue, *S. meliloti*; green, *Brucella melitensis*; and yellow (or brown) *M. loti*. B, frequency distribution curves for the %chSp of the five organisms. Values above 50% were omitted for clarity. Color lines as in A, with exception of brown for *M. loti*. C, functional distribution of orthologs by species signature. Data from *R. etli *products. Functions were classified into Metabolism (red line, including amino acid, nucleotide, fatty acid, and cofactor biosynthesis, central intermediary metabolism, and energy generation), Information (green line, DNA metabolism, transcription, translation, and transcriptional regulators), Processes (blue line, transport; cellular envelope synthesis, and other cellular processes), and Hypothetical functions or poorly characterized proteins (orange line).

With the %chSp we wanted to find the specific differences in protein sequences of the orthologs for each of the species studied. The %chSp was calculated by normalizing the number of residues in the ortholog sequence that occur only once in individual alignment columns [4]. This measure denotes the amount of most specific residues in the orthologs, and is expressed as a percentage of the total number of amino acids in the proteins. In Figure 1A, the %chSp of the orthologs studied is shown. Two groups can be clearly distinguished, with *S. meliloti *grouped with *A. tumefaciens *and *R. etli*, and *M. loti *and *B. melitensis *in another group. That grouping reflects the phylogenetic relationship of these species. Frequency distribution curves of the %chSp values were plotted to illustrate in detail the separation of these groups (Figure 1B).

To detect the effect of divergence on functional role, we plotted the distribution of functional classes by the %chSp. As observed in Figure 1C (for *R. etli*, the other species gave a similar pattern, not shown), the informational functions (replication and DNA metabolism, transcription, translation, and transcriptional regulation) showed the most reduced amount of the %chSp, followed by metabolic functions, and cellular processes. The hypothetical functions or poorly characterized proteins showed the highest level.

**Figure 2:**
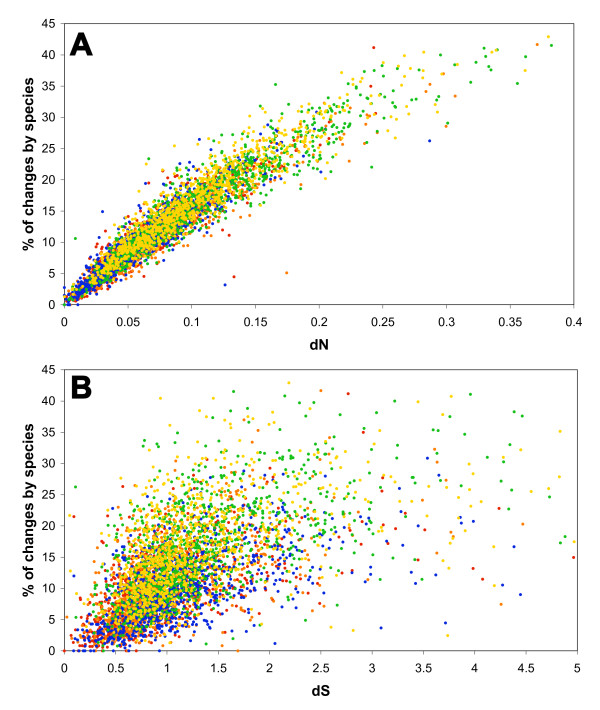
**Relationship of the percentage of changes by species (%chSp) of orthologs with synonymous (dS) and nonsynonymous (dN) substitution rates**. A, relationship of dN with the %chSp. B, relationship of dS with the %chSp. dN and dS values were obtained with PAML [22]. Dot colors: orange, *R. etli*; red, *A. tumefaciens*; blue, *S. meliloti*; green, *B. melitensis*; and yellow, *M. loti*.

To determine the usefulness of the %chSp, we compared this parameter with the codon evolution of the orthologs. To do this, we calculated the synonymous (dS) and nonsynonymous (dN) substitution rates by the maximum likelihood method with PAML [22]. Some genes showed dS saturation and were eliminated from the analysis; 985 genes remained with dS values lower than 5. We obtained very high correlations between the %chSp and dN (Figure 2A) and dS (Figure 2B), with Pearson coefficients of r = 0.932 to 0.953 (with p < 0.05) for relation with dN, and r = 0.517 to 0.587 (p < 0.05) for dS. These data revealed that the %chSp was well correlated with codon evolution and is a useful tool to extract directly the evolutive change at the protein level. In comparison, other measures of sequence conservation, such as protein identity or similarity, gave lower Pearson coefficients with dN or dS (for example, in *S. meliloti*, dN-common identity r = -0.798, dN-common similarity r = -0.650; dS-common identity r = -0.511, dS-common similarity r = -0.400) (not shown). dN and the %chSp are related measures, but they are not derived from the same sequence information: dN is calculated from nonsynonymous codons in nucleotide sequences, while the %chSp is calculated directly from protein sequences by extracting the most specific residue for each homologous position after sequence alignment.

**Figure 3:**
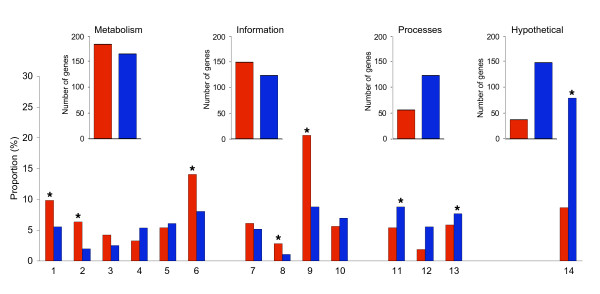
**Functional distribution of orthologs divided by low or high nonsynonymous substitution rate (dN)**. The graph was generated using 985 orthologs with dS < 5; they were divided as described in the Methods section. Functions were grouped into Metabolism (1, amino acid biosynthesis; 2, nucleotide biosynthesis; 3, fatty acid biosynthesis; 4, cofactor biosynthesis; 5, central intermediary metabolism; and 6, energy generation), Information (7, DNA metabolism; 8, transcription; 9, translation; and 10, transcriptional regulators), Processes (11, transport; 12, cellular envelope synthesis; and 13, other cellular processes), and 14, Hypothetical functions. Bar colors: red, orthologs with low dN; blue, orthologs with high dN. Asterisks denote significant differences with p < 0.05, Fisher's exact test.

Based on values obtained from each of the five species (see Methods section), the orthologs were classified into groups either with low or high dN: 426 were classified as having a low dN value, and 559 as having a high dN value. A functional distribution was generated and, significantly, metabolism (amino acid and nucleotide biosynthesis and energy generation), and informational (transcription and translation) functions tended to be encoded by genes with low dN values (Figure 3, significant difference by Fisher's exact test, with p < 0.05). In contrast, genes belonging to the high dN group had significant abundance of hypothetical functions, and also contributed particularly to cellular processes (transport and cellular envelope). The significance of these differences is explained in the Discussion section.

### Phylogenetic relationships of orthologs

To detect the phylogenetic relationships in the orthologs under study, we obtained their phylogenetic trees by maximum likelihood with Phylip [21]. Three main topologies were found. The first, which included 52% of the orthologs, associated *R. etli *with *A. tumefaciens *in the same branch (denoted as *R. etli-A. tumefaciens*, *S. meliloti*, *Brucella melitensis-Mesorhizobium loti *or a RA-S-BM topology, for the initials of the species); the second, comprising 24%, joined *R. etli *with *S. meliloti *(RS-A-BM topology); and the third, with 13%, connected *A. tumefaciens *with *S. meliloti *(AS-R-BM topology). The remaining possible tree combinations accounted for 11% of orthologs. A phylogenetic marker, such as 16S rRNA supports the most abundant phylogeny RA-S-BM, in agreement with a closer association of *R. etli *and *A. tumefaciens *(Additional file 1) [26]. Other markers, such as AtpD (RS-A-BM), RecA (RA-S-BM) and DnaJ (RA-S-BM) were also found distributed in the main phylogenies.

### Physico-chemical properties of the ortholog products

We applied the five series of values reported by Atchley *et al. *[9], to determine how the sequence variability of the orthologs affects the biochemical characteristics of the encoded proteins. Each series of values analyzes a special feature of the residues, namely polarity, secondary structure, volume, amino acid composition, and electrostatic charge [9]. The calculated values of the ortholog products spanned a wide range, from negative to positive values (with the exception of the amino acid composition, which only gave positive values). The electrostatic charge and volume showed the best correlation values (Pearson coefficient r = 0.848 in *S. meliloti*), followed by volume and amino acido composition (r = -0.662). We detected an atypical group of 110 protein sequences in polarity (for example, in the comparison with amino acid composition), consisting almost entirely of proteins with a high number of transmembrane segments (data not shown). The low polarity of membrane proteins has long been known [27], confirming the appropriate application of the method. The rest of the organisms showed similar tendencies (not shown).

**Figure 4:**
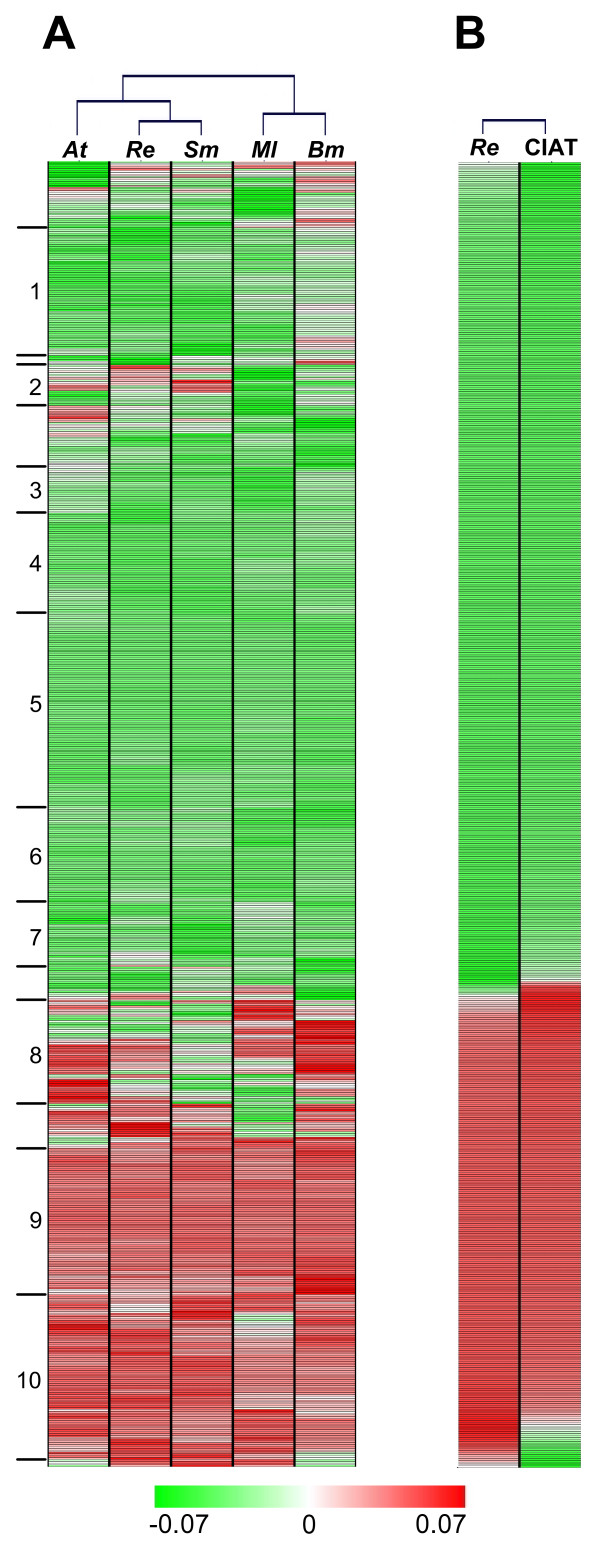
**Association among species by physico-chemical characteristics of ortholog products**. A, polarity is shown as example. The panel shows 705 syntenic ortholog products per species. Letters at the top: *At, A. tumefaciens; Re, R. etli; Sm, S. meliloti; Bm, B. melitensis; *and *Ml, M. loti*. Lines at left denote clusters of orthologs with similar profile in the five species (ten were selected and analyzed in the text). B, comparison of two *R. etli *strains with polarity. The panel shows 705 syntenic ortholog products per strain. Letters at the top: *Re, R. etli *strain CFN42; CIAT, *R. etli *strain CIAT652. Clustering was performed with MeV TM4 [23]. Scales for normalized values (green for negative values, white for zero, and red for positive).

With a clustering method [23], we tried to find relationships among species derived from the physico-chemical nature of the residues encoded by the orthologs. We generated clustering profiles for each property (shown for polarity, Figure 4A, and the rest in the Additional file 2) with the most conserved gene set (705 syntenic orthologs with dS < 5). A high level of variation among each ortholog can be observed, reflecting specific changes in each of the species. For polarity, a closer association of *R. etli *with *S. meliloti*, followed by *A. tumefaciens *in another branch, was observed (an RS-A-BM association, Table 1); this relationship was similar to the second most abundant phylogeny described above. For the rest of the properties, other associations were observed: RA-S-BM for secondary structure, electrostatic charge, and volume (Additional file 2 and Table 1), and again RS-A-BM for amino acid composition (Table 1). These associations revealed which physico-chemical character links most closely with the species, reflecting the phylogenetic relationship and also the lifestyle of the organisms. When the complete 1416-ortholog set was analyzed, *B. melitensis *departed clearly from the rest of species in polarity, charge and amino acid composition (Table 1, denoted as M-B in independent branches). In the group of Rhizobiales analyzed, *Brucella *is the only non free-living organism.

**Table 1:** Relationship of phylogeny and species association by physico-chemical property.

		Association by physico-chemical property				
**Ortholog classes**	**Number of genes**	**Polarity**	**Secondary structure**	**Volume**	**Amino acid composition**	**Electrostatic charge**

Orthologs	1416	RS-A-M-B	RS-A-BM	RA-S-BM	RS-A-M-B	RA-S-M-B
Syntenic orthologs	705	RS-A-BM	RA-S-BM	RA-S-BM	RS-A-BM	RA-S-BM

Syntenic orthologs divided by phylogeny						
RA-S-BM	380	RS-A-BM	**RA-S-BM**	**RA-S-BM**	RS-A-BM	**RA-S-BM**
RS-A-BM	180	**RS-A-BM**	**RS-A-BM**	RA-S-BM	**RS-A-BM**	RA-S-BM
AS-R-BM	92	RS-A-BM	**AS-R-BM**	RS-A-BM	RA-S-BM	**AS-R-BM**

Syntenic orthologs with phylogenies other than the main three shown (accounting for 53 products) were discarded from the analysis. Letters denote species: R: *R. etli*, S: *S. meliloti*, A: *A. tumefaciens*, B: *Brucella melitensis*, M: *Mesorhizobium loti *(a pair of letters denote that these species are in the same branch). Species association remarked in bold denotes equivalence with phylogeny.

For a more detailed study of niche association by physico-chemical properties, we obtained the clustering for 17 alpha-proteobacterial species and 23 proteins, mainly from metabolism. The species group covered plant-associated organisms (*Methylobacterium*, *Azospirillum*), plant symbionts (the rhizobia), animal (*Bartonella*, *Anaplasma*) and plant (*Agrobacterium*) pathogens, and free-living species (*Caulobacter*, *Xanthobacter*, *Oligotropha*, among others). The most clear cluster associations were for amino acid composition (with clusters containing only symbionts, free-living species, or pathogens), volume and electrostatic charge (with clearly defined clusters for symbionts or pathogens) (Additional file 3). In contrast, polarity and secondary structure showed a majority of phylogenetic associations.

To search for the relationships of physico-chemical associations with phylogeny, we determined the associations of each group of orthologs with a given phylogeny. We analyzed the most conserved group, 705 syntenic orthologs. In the case of 380 orthologs with phylogeny RA-S-BM, the same association was obtained for secondary structure, volume, and charge (Table 1). The orthologs with phylogeny RS-A-BM produced the same association for secondary structure, polarity, and amino acid composition. The orthologs with the phylogeny AS-R-BM showed the same association for secondary structure and charge (Table 1). These data showed that secondary structure was the physico-chemical property most consistent with phylogeny (because each phylogenetic group showed the same association in that property); in contrast, for polarity, all groups reflected the same association (RS-A-BM), indicating a strong coherence of that property. For the rest of properties, the main phylogenetic groups (by number of orthologs, RA-S-BM or RS-A-BM) dominated the species association.

We analyzed in more detail groups of products from the polarity clustering (Figure 4A). Ten clusters were selected, comprising 83% of the total orthologs under analysis. Clusters 1, 4, 5, and 7 presented abundance in metabolic functions, while clusters 9 and 10 were abundant in informational functions. In concordance to their profile homogenity (red or green in Figure 4, positive or negative signs in the table of the Additional file 4), their members showed reduced levels of the percentage of changes by species (%chSp) (Additional file 4). A cluster with more diverse levels, such as 8, contained proteins pertaining to hypothetical functions, with high values of %chSp (Additional file 4).

In order to have an assessment at the strain level, we compared the chromosomal orthologs of *R. etli *CFN42 with *R. etli *CIAT652, which shows better symbiotic performance than CFN42 [18,28]. We found an almost identical pattern of polarity between the two strains, but also found small groups of products with diverse values (Figure 4B), with a significant abundance of products belonging to the functional classes of cell wall synthesis, DNA metabolism and cellular processes (especially motility) (not shown).

**Figure 5:**
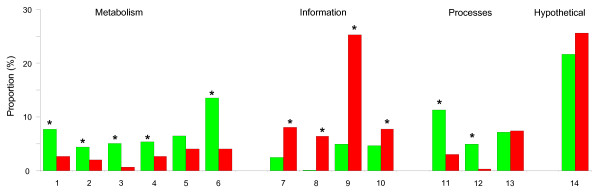
**Relationship between function and sign of values of ortholog products obtained with polarity**. From left to right, proportions of functional classes grouped into Metabolism (1, amino acid biosynthesis; 2, nucleotide biosynthesis; 3, fatty acid biosynthesis; 4, cofactor biosynthesis; 5, central intermediary metabolism; and 6, energy generation), Information (7, DNA metabolism; 8, transcription; 9, translation; and 10, transcriptional regulators), Processes (11, transport; 12, cellular envelope synthesis; and 13, other cellular processes), and Hypothetical functions (14). Bar colors: green, negative; red, positive. Asterisks denote significant differences with p < 0.05, Fisher's exact test.

Given the observed functional tendencies described above, we divided the products having the same sign in the five species and obtained functional distributions. We found an interesting relationship between the functional role of the protein and its sign from the physico-chemical property evaluation, which could be related to specific biochemical requirements for functional performance. For polarity, functions of metabolism and processes were related to negative signs, while informational functions were strongly related to positive signs (significant differences with p < 0.05, calculated with Fisher's exact test) (Figure 5). Several other remarkable features were found: for secondary structure, the negative sign (related to abundant alpha-helical structures) was associated with informational functions, and the positive sign (abundant coils) was related with synthesis of the cellular envelope and hypothetical proteins (Additional file 5). Hypothetical proteins showed significant abundance of low level amino acid composition (i. e., atypical composition); translation was abundant in protein sequences with positive charge; and energy and translation functional classes showed residues with high molecular volume (Additional file 5).

To find a relationship between the physico-chemical characteristics of proteins and evolutionary rates, we plotted distribution curves for gene groups with low or high dN and their physico-chemical values, for each of the properties. A striking general tendency was observed, because low dN genes presented significant abundance of sequences with positive sign in polarity, volume, and electrostatic charge (Additional file 6, significant differences in distribution curves, Mann-Whitney test, with p < 0.05), revealing a specific *physico-chemical *evolution of products.

**Figure 6:**
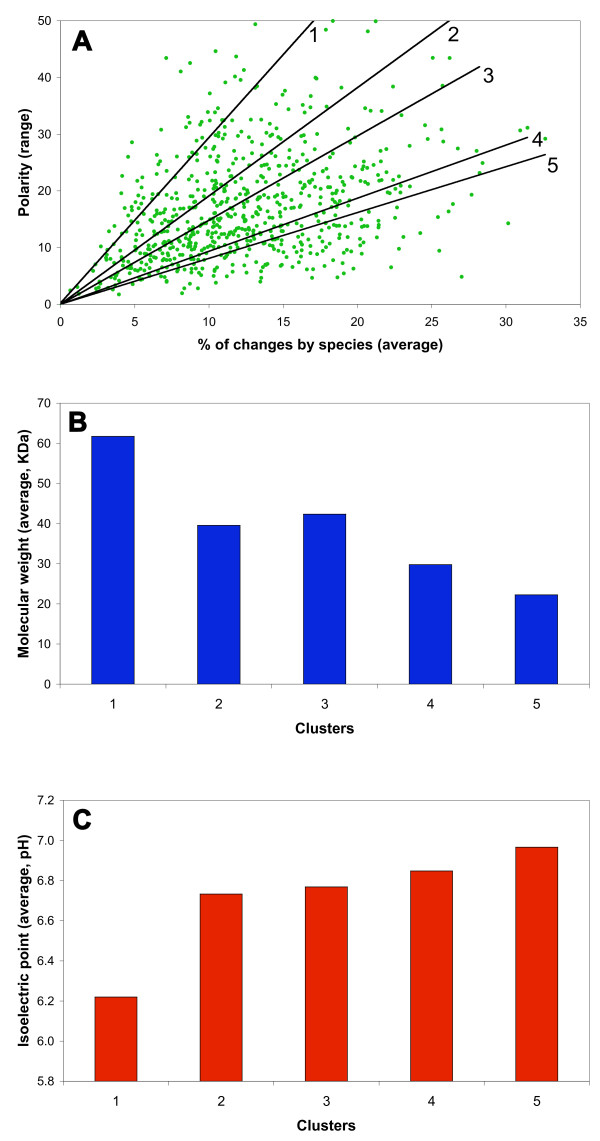
**Relationship between the physico-chemical properties and the percentage of changes by species (%chSp)**. A, dot plot graph of the average %chSp and polarity (range). Tendency lines for the five clusters are shown. B, molecular weight (average) of syntenic ortholog products, following the order of the slope of clusters (up to down). C, isoelectric point (average) of syntenic ortholog products, following the order of the slope of clusters.

To determine the relationship of the %chSp with physico-chemical properties, we generated a dispersion graph, considering the average of the %chSp and the range of polarity, as shown for example in Figure 6A. A positive relationship was obtained, revealing that sequence variability corresponded with diversity in the character evaluated (a similar tendency was observed in the rest of the properties, not shown). The dispersion was analyzed by obtaining clusters with high correlation values (see Methods). Strikingly, clear tendencies in molecular weight and isoelectric point were obtained. The molecular weight was progressively reduced according with a negative slope (Figure 6B), while the opposite was observed for isoelectric point (Figure 6C). Similar tendencies were observed in the three other properties, with the exception of electrostatic charge (not shown). The first cluster had significant abundance of proteins from the functional classes of amino acid biosynthesis, transcription and energy generation (Fisher exact test, p < 0.05), and the last one was abundant with proteins of hypothetical functions and some cellular processes (not shown).

Lastly, we analyzed the data for a specific syntenic ortholog, *argC*, the gene that we used previously to experimentally validate the %chSp approach. We reported the experimental effect of ortholog replacement in an *S. meliloti argC *mutant [29]. The *argC *from *M. loti *showed almost a complete inability to functionally complement the native gene in minimal medium supplemented with succinate-ammonium or mannose and nitrate [29]. The *M. loti *product showed the most differences in volume, secondary structure, and electrostatic charge, in comparison with *S. meliloti *(in the table of the Additional file 7). Possibly these three physico-chemical properties were important to define the phenotypic behavior of *M. loti *ArgC complementation. *R. etli *and *A. tumefaciens *genes, with similar divergence to *S. meliloti*, showed a better complementation phenotype but, interestingly, they also showed some differences. The *A. tumefaciens *gene produced a reduced growth ability and exhibited a strong excretion of metabolites, resembling the *argC *mutant strain [29]. The *A. tumefaciens *product was more similar to that of *M. loti *in charge and volume (Additional file 7). On the other hand, the *R. etli *gene showed a growth phenotype most resembling that of *S. meliloti*, and the products from *R. etli *and *S. meliloti *had identical values in volume, and some similar values in charge; therefore, these properties can be considered as the most important physico-chemical factors in determining the observed complementation efficiency.

## Discussion

Several studies have focused on essential gene recognition and evaluation of evolutionary rates [30-36], but few studies in bacteria have analyzed the relationship of evolutionary rates and phylogeny with the functional role and the physico-chemical properties of a massive group of proteins. We used new parameters to evaluate these traits, including the percentage of changes by species (%chSp), and a series of values for physico-chemical characteristics of amino acid residues; also, we consider our group of orthologs (mainly syntenic genes), which perform housekeeping functions and also a diversity of cellular tasks, as an adequate gene set to study the conserved characteristics among related species, but also to analyze the ortholog variability and relationship with function and niche adaptation.

We analyzed 7080 genes (organized in 1416 sets of five values each) evaluating how their sequence variability could affect the physico-chemical characteristics of the encoded proteins. Previously, we proposed a parameter, the %chSp, to extract species-specific information that may be related to the cellular milieu and niche adaptation [4]. We showed the values and distribution of the %chSp of the chromosomal orthologs (two groups of species were formed), and how the functional classes showed differential amounts of %chSp (Figure 1). Strikingly, we found a very high correlation of this parameter with codon evolution (Figure 2), mainly nonsynonymous substitution rate (dN), indicating that the %chSp is an efficient way to obtain important evolutive information directly from the protein sequence. Some homologous positions in multiple alignments of proteins were reported earlier to be associated with functional variation [37].

Most importantly, non-neutral variations in genes can change the potential of the cell for further adaptations [5,38-41], and even neutral modifications, as explained in the frame of the constructive neutral evolution, about fixation of neutral or slightly deleterious mutations [42]. In our case, protein sequences of orthologs presented different values in each of the five physico-chemical series of values (Figure 5 with polarity as example, and more pronounced in the rest of properties, Additional file 5), that reflected the vast amount of specific changes in each ortholog and species, possibly responding again to the intracellular milieu and niche adaptation. In the comparison of orthologs belonging to *R. etli *strains [18,28], very few changes were observed (polarity as an example, Figure 4B), demonstrating the overall closeness of these organisms. We propose that variability of orthologs determine key properties of the proteins. We found that changes in orthologs indeed define particular physico-chemical characteristics. In this sense, these findings indicate that changes in orthologs (mainly nonsynonymous substitutions), defined commonly as neutral [5,43], determine particular physico-chemical characteristics that lead to alterations and represent functional requirements. In this context, it was found that most of the mutations in ribosomal protein genes were similar and weakly deleterious but, mostly important, the distribution of fitness for synonymous and nonsynonymous mutations were similar [44].

In an experimental approach in our laboratory, we substituted an essential syntenic ortholog (*argC*) from the arginine biosynthetic pathway of *Sinorhizobium meliloti*, and expressed *argC *orthologs from other Rhizobiales in that background, with the aim of demonstrating the relationships of particular residues with function and evolution. By a physiological, enzymatic, transcriptional, and proteomic characterization we found that several factors, such as the type of promoter and the percentage of changes by species, determined different functionality and levels of transcription and translation of the ArgC protein [29]. We used the physico-chemical series of values obtained by Atchley, to correlate the physico-chemical properties of the proteins with the real phenotype and performance of *argC *orthologs, in a *S. meliloti *background. As observed in Additional file 7, the *R. etli *ortholog, with more similar physico-chemical values to *S. meliloti*, showed the most resembling phenotype; followed by the *A. tumefaciens *gene, the *M. loti argC *showed a severe growth deficiency and very different physico-chemical values, in comparison with *S. meliloti*. As Dr. Koonin and collaborators said regarding our work [45], it is necessary to consider that functional conservation among orthologs should be inferred with caution because some orthologs genes can diverge functionally even among closely related organisms. Also, there are other factors that influence adaptation in bacteria, such as compensatory gene amplification after orthologous replacement [46], which was also found in our work.

Most essential functions, such as information and metabolism were significantly covered by low dN genes in the five species, showing more restriction to evolution; in contrast, orthologs with high dN were functionally more flexible, abundant in hypothetical functions and cellular processes, but perhaps also showed more dispensability (Figure 3). The differences in evolutionary rates of genes have been adscribed to functional constraints, but a debate is open to clarify the importance of translational costs, reflected in misfolding [47], structural factors [48,49], and even pleiotropy [50], as the factors influencing the evolution of proteins. The compositional and adaptation codon bias in genes are important characteristics of GC-rich organisms, such as the Rhizobiales, because these traits are related to increased expressivity [51-53]. We found that low dN genes had high CAI values (not shown) showing, as others have, that codon bias is a selective force in these genes and a negative correlation of expressivity and evolutionary rates exists [54-56].

We found that the series of values reported by Atchley [9] were very useful to discern the contribution of physico-chemical factors of amino acids to the characteristics of the ortholog products. These values summarize the amino acid features into five biochemical characteristics, namely polarity, secondary structure, amino acid composition, molecular volume, and electrostatic charge [9]. We found the global correlations among the physico-chemical properties that can be possibly generalized to all bacterial proteins. Interestingly, we found a group of products, clearly differentiated from the rest by polarity, composed almost exclusively by proteins with many transmembranal segments (not shown). This result confirmed that these series of values can detect specific features of residues forming part of proteins. Recently, Marsella *et al. *[57], using the series of values reported by Atchley detected an especial periodicity of solenoid secondary structures. In another study, the same series of values were used to define niche adaptation of prokaryotic organisms from extreme environments [58].

When orthologs were related by their physico-chemical features, species associations were similar to the most abundant phylogenies, indicating a link between characteristics of proteins and species evolution (Figure 4A, and Additional file 2); however, they also showed differences that may account for other structural factors related to intra- and extra-cellular environments in shared niches, for example *R. etli *products most closely related to those from *S. meliloti *in polarity and amino acid composition (possibly because both organisms are symbionts), but most closely to *A. tumefaciens *products in secondary structure, volume, and electrostatic charge (in this case, close phylogenetically relatedness can be argued). These relationships shed light about the importance of considering an approach such as that described here. In this regard, in comparison with classical phylogeny, the physico-chemical approach of species association can give an additional amount of information. *B. melitensis *orthologs, despite their closer phylogenetic relationship with *R. etli*, *S. meliloti *and *A. tumefaciens*, departed from the other four species in amino acid composition, charge and polarity (not shown), possibly reflecting differential adaptation in this set of orthologs; *Brucella *is the only species of the analyzed with a non free lifestyle. Recently, it was reported that *B. melitensis *has a more basic proteome than the rest of Rhizobiales, possibly in response to its intracellular lifestyle [59], in addition to being subjected to genomic erosion processes (such as gene loss, pseudogenization, and reduction of CAI), like other intracellular organisms [60-63]. In the clustering analysis covering many alpha-proteobacterial species and their typical lifestyles, clear niche associations were found for some properties (Additional file 3).

A remarkable result was the observation that orthologs with low nonsynonymous substitution rate (dN) were associated with specific amino acid characters, such as higher polarity and electrostatic charge, and large molecular volume (Additional file 6), possibly reflecting physico-chemical tendencies of the evolution of genes. Additionally, the functions of orthologs were related with the particular sign of values from physico-chemical property evaluation (Figure 5, and Additional file 5), indicating more or less strict specific biochemical requirements of the residues in the proteins to perform their role. In some cases, there were clear differences between the metabolic and informational functions, or between the metabolic and processes functions. Furthermore, some of the hypothetical protein sequences appeared with the same sign as that of metabolic genes or genes encoding proteins involved in processes; the qualification with these series of values would be useful in the eventual functional characterization of the hypothetical proteins. Additionally, interesting tendencies were found regarding molecular weight and isoelectric point, along with the increase of the percentage of changes by species (Figure 6). These findings clearly merit a more detailed analysis.

## Conclusions

In this work, we found that the sequence variability of the chromosomal orthologs of the Rhizobiales species under study have evolutionary and functional implications, closely related to the physico-chemical properties of the products. We found that the percentage of changes by species of the orthologs directly reflects the nonsynonymous substitution rate; and that there was a relationship of the physico-chemical characteristics of residues encoded in the proteins with evolution and specific functional requirements. This genomic study contributes to the understanding of factors that connect the evolution of genes with the physico-chemical characteristics of the proteins and their functional adaptation.

## Competing interests

The authors declare that they have no competing interests.

## Authors' contributions

JM and HP conceived of the project. HP, GG, AA, and JM performed the analysis and analyzed the results. HP and JM wrote the draft manuscript. All authors read and approved the final manuscript.

## Reviewers comments

### Reviewer report 1. Dr. Purificación López-García, Centre National de la Recherche Scientifique, France

Peralta and co-workers present a comparative analysis of 1416 orthologous proteins shared by five species of Rhizobiales, three plant symbionts, one plant pathogen and one animal pathogen. After carrying out phylogenetic analyses of those orthologs and finding three major topologies, Peralta et al. study the sequence variability of a subset of these proteins by looking at the number of synonymous (dS) and non-synonymous (dN) substitutions and a related measurement, the "species signature" defined as the number of species-specific amino acid residues. The authors study possible correlations between protein sequence variability and a series of associated properties (polarity, electrostatic charge, secondary structure, molecular volume and amino acid composition) and find particular trends, which suggest that particular physico-chemical properties are associated with particular functional roles. The authors further speculate that much sequence variation is not related to phylogeny but to adaptation to particular niches. Although the last conclusion needs further testing, the overall work is properly done and interesting and I have only some relatively minor comments.

Author's response: Thanks for your commentary. Effectively, the question about how much of the divergence belong to neutral variation and what amount to adaptation is our departure point for the analysis. For a long time, we have wondered about how to disect the problem. We consider that our approach is useful to analyze in detail the sequence variability and we are beginning to find answers about this issue. Please, also see our response for the last question.

My first comment relates to the "species signature". The authors find "striking" to see a "correlation of this parameter with codon evolution, mainly nonsynonymous substitution rate (dN), indicating that the species signature is efficient to obtain important evolutive information, directly from the protein sequence". This is not striking at all since, even if dN is estimated from DNA sequence, it corresponds in fine to changes in amino acid sequence, so that the "species signature" is but a subset of the amino acid changes resulting from non-synonymous substitutions (dN). It is normal that they both contain evolutionary information because the two measures are correlated. What I do not understand well is the advantage of the "species signature" versus dN. The number of non-synonymous substitutions is independent of taxonomic sampling, but the "species signature" depends on taxonomic sampling. By contrast to dN, as more and more strains or species genome sequences become available within a genus, the "species signature" will most likely progressively decrease and eventually reach zero (especially if one considers orthologs for a same protein from the whole tree of life). In this sense, the use of the "species signature" seems limited and restricted to particular case studies.

Author's response: We eliminated the word "striking" in the beginning of the mentioned paragraph. Our proposal of species signature is devoted to distinguish certain differences in closely related genomes. The signature reaches zero when sequences from organisms show less phylogenetic differences; i. e., ideally when they form part of the same species.

The authors show that changes in orthologs correlate with particular physico-chemical properties of the corresponding proteins and, hence, likely functional adaptation to particular niches. However, this correlation remains at a theoretical, predictive level. Since the studied proteins are annotated and their functions known, it would be very illustrative if the authors could point at one or several particular examples and explain them in the context of their analysis.

Author's response: With the last analysis in the Results section, we tried to relate the phenotypic experimental differences of the argC complementing strains with its physico-chemical properties.

As stated in the Discussion section, even when each ortholog performs the same function in a given genome, without doubt the gene (and its product) must be adapted in order to respond to a particular physiological environment.

Although the mentioned proposal seems speculative, it is important to take into account our recent publication (Díaz et al., J. Bacteriol. 193:460-472, 2011), in which our results from complementation experiments showed that the substitution of the S. meliloti argC gene, with the corresponding ortholog from other Rhizobiales species, was not completely functional, and also affected the growth, transcription rate and translation, and possibly the whole cellular physiology. Indeed, as Dr. Koonin said recently of our work (Kristensen, et al., Brief. Bioinf., 2011, doi:10/1093/bib/bbr030), it is necessary to consider that functional conservation among orthologs should be inferred with caution, because some orthologous genes can diverge functionally even among closely related organisms. As a matter of fact, this manuscript was the initial approach that allowed us to design the experiments of the already published work.

### Reviewer report 2. Prof. Jeffrey Townsend (nominated by Prof. J. Peter Gogarten), Yale University, United States

The revised manuscript clarifies the "species signature" metric used, and addresses issues related to its comparison to dN fairly satisfactorily. The manuscript does not improve or extend its results by applying either of the two approaches recommended that would considerably empower the conclusions being drawn.

From the original review:

First, the relationships between physico-chemical characteristics of residues encoded and functional category of the gene could be analyzed with more specific and powerful statistical approaches. In particular, instead of dividing a continuous range arbitrarily into two discrete categories to perform purely categorical statistical analyses, a more powerful approach would consist of performing logistic regressions with the continuous variable on the x-axis and the categorical on the y (i.e. high or low dN as a continuous predictor of functional category). Second, the manuscript addresses gene-wide evolutionary rate and correlates it to gene-wide physico-chemical metrics. Because each of these metrics is "averaged" across a gene, considerable power to detect important signal is lost. A more direct way to assess these questions would be to correlate evolutionary rates of individual sites and physico-chemical properties of those sites. Examining the rates of evolution of sites and of physico-chemical properties of amino acids could also better clarify the phylogenetic discrepancies among orthologs noted in this manuscript. These discrepancies among inferences from the orthologs are noted, but are attributed without much detail to selection based on ecological niche, and are not directly evaluated in terms of potential phylogenetic signal, noise, or horizontal gene transfer of the gene sequences, all of which may play roles in the different results achieved when analyzed solo for phylogenetic inference.

### Reviewer report 3. Ms. Olga Kamneva, University of Wyoming, United States

The authors analyzed the set of orthologous gene families from five strains of five different species of Rhizobiales using several techniques, the authors aim to characterize patterns of sequence evolution in relation to physical properties of encoded proteins within gene families and examine how change of protein physicochemical properties is related to niche adaptation.

I think that while the authors had an interesting and novel idea, to investigate how the physical properties of evolutionary related proteins are changed across related organisms of distinct life styles I feel that quality of the study can be significantly improved by shifting the focus of the study from trying to find correlation between change of physical properties and evolutionary rates to characterizing mechanisms and consequences of change of the physical properties. I also have some technical comments.

Specific comments:

1. The study should benefit from using one of the clustering based approaches to identify gene families with subsequent inference of orthologs. Using bidirectional best hit for this leads to under-determination of inparalogs within orthologous groups, as well as including xenologs into the gene families.

2. The study would also benefit from using modern phylogeny inference software (PhyML or RaxML), the results should not differ much but will be more robust.

3. A dS threshold value of 5 is too high, dS values larger than 3 is considered to be a sign of synonymous sites saturation even when estimated using maximum likelihood [Z. Yang, personal communication http://gsf.gc.ucdavis.edu/viewtopic.php?f=1&t=2291&p=3967&hilit=saturation&sid=224cf845cae40709bad44196fb33ffed#] Also the threshold should be applied to the depth of the whole tree, not just certain branches. If synonymous sites are saturated dS will be determined wrong and it does not matter what it is used for, dN/dS calculation or to see how well dS correlates with something else (not dN).

4. The authors use "percentage of changes by species" as measure of species-specific protein sequence variation. As it is explained in the manuscript and follows from the name of the metric, "percentage of changes by species" is a percentage of the protein alignment columns where the sequence from one particular species has a different residue than the rest of the alignment column. Basically "percentage of changes by species" is a crude approximation of the number of amino acid substitutions that occurred on a branch leading to the sequence over the total length of the sequence. The only benefit of the used metric is the ease of calculation, while terminal branch length of protein gene trees (or dN trees) appears to be an estimate of the same thing and be technically more rigorous. I suggest not using "percentage of changes by species" for this study at all.

5. Figure 2 in my opinion should be omitted as well as use of "percentage of changes by species".

6. I like the idea of clustering organisms based on the physical properties of proteins in gene families (Figure 5 and Additional file 3). Correlation between life style and proteome properties was shown before [Tekaia F et al. Gene. 2002.], ref [58,59] of the manuscript, while underlying biology of these observed patterns is unknown. Looking at changes of physical properties of evolutionary related proteins from organisms of different life style is novel. However with only one animal pathogen and one plant pathogen in the set (Figure 5), it is not possible to recover something meaningful while on the Additional file 3 only clustering based on amino acid composition is clear. Thus I am not sure if conclusion about correlation of protein properties and species ecology has much of support.

7. Figure 5 shows proteins which are extremely polar in one genome and extremely non-polar in another. It is interesting to see how this shift in polarity or any other property occurs. Is it caused by an additional domain which perturbs the average or it is due to point mutations? It is also interesting to see how this shift affects structure/function of the proteins. 1-2 case studies should be shown (alignments, comparison of protein structures, conservation/alteration of functionally important residues). It would be a first attempt to reveal molecular mechanisms underlying the correlation between ecology of microbes and properties of their proteomes.

## Supplementary Material

Additonal file 1 **Phylogenetic tree of 16S rRNA genes**. Color dots denote lifestyle: green, symbionts; red, pathogens. The tree was obtained with Phylip. Bar, substitutions per nucleotide. Click here for file

Additional file 2 **Association of species by physico-chemical characteristics**. Clustering was performed with MeV TM4 [23], with Pearson correlation. Each panel shows 705 syntenic ortholog products per each of the five species. Letters: *At (A. tumefaciens), Re (R. etli CFN42), Sm (S. meliloti), Ml (M. loti)*, and *Bm (B. melitensis)*. Scales for normalized values (green for negative values, white for zero, and red for positive). Click here for file

Additional file 3 **Association of alpha-proteobacterial species by physico-chemical characteristics**. Clustering was performed with MeV TM4 [23], hierarchical Pearson correlation, using 23 protein sequences and 17 species. Square color in name denotes lifestyle: green, plant-associated and symbiotic species; blue, free-living organisms; red, animal and plant pathogens. Left, 16S rRNA gene tree phylogeny, obtained with Phylip. Right, association of clusters from physico-chemical properties, as follows: A, amino acid composition; B, volume; C, electrostatic charge; D, secondary structure; and E, polarity. Protein sequences: ArgB, AtpA, AtpB, AtpC, AtpD, DnaJ, DnaK, FabD, FabH, NuoA, NuoB, NuoC, PurB, PurD, PurF, Tmk, AcnA, GltA, Mdh, Pgk, SdhA, SucA, and SucB. Organisms: Plant symbionts and plant-associated bacteria, additional to *R. etli *(ret)*, S. meliloti *(sme), and *M. loti *(mlo): *Bradyrhizobium japonicum *UT26S (designated as bja, GenBank accession no. NC_014013.1), *Azorhizobium caulinodans *ORS571 (azc, NC_009937.1), *Methylobacterium extorquens *AM1 (mea, NC_012808.1), *Azospirillum *sp. B510 (azl, NC_013854.1). Free living species: *Xanthobacter autotrophicus *Py2 (xau, NC_009720.1), *Rhodopseudomonas palustris *BisA53 (rpe, NC_008435.1), *Oligotropha carboxidovorans *OM5 (oca, NC_015684.1), *Nitrobacter winogradsky *Nb-255 (nwi, NC_007406.1), *Caulobacter crescentus *CB15 (ccr, NC_002696.2). Animal and plant pathogens, additional to *A. tumefaciens *(atu) and *B. melitensis *(bme): *Ochrobactrum anthropi *ATCC 49188 (oan, NC_009668.1), *Bartonella henselae *Houston-1 (bhe, NC_005956.1), and *Anaplasma centrale *Israel (acn, NC_013532.1). Bars denote substitutions per nucleotide (16S rRNA tree) and arbitrary distances (physico-chemical properties), respectively. Click here for file

Additional file 4 **Characteristics of selected groups of products from clustering of polarity**. Click here for file

Additional file 5 **Relationship between function and sign of values of ortholog products obtained with the series of values of physico-chemical properties**. In the top row (from left to right), number of orthologs grouped into Metabolism (1, amino acid biosynthesis; 2, nucleotide biosynthesis; 3, fatty acid biosynthesis; 4, cofactor biosynthesis; 5, central intermediary metabolism; and 6, energy generation), Information (7, DNA metabolism; 8, transcription; 9, translation; and 10, transcriptional regulators), Processes (11, transport; 12, cellular envelope synthesis; and 13, cellular processes), and Hypothetical functions (14). In the bottom row, the functional classes in proportions. Asterisks denote significant differences with p < 0.05, Fisher's exact test. Bar colors: green, negative (low level for amino acid composition); red, positive (high level for amino acid composition). Scale for amino acid composition graph was broken to conserve the shape. Click here for file

Additional file 6 **Relationships between the nonsynonymous substitution rate (dN) and the physico-chemical properties**. Frequency distribution curves of the orthologs divided in groups with low or high dN values. A, polarity. B, volume. C, electrostatic charge. Data from *S. meliloti *products. Low dN group, orange line. High dN group, green line. Click here for file

Additional file 7 **Values of physico-chemical properties for ArgC from Rhizobiales**. Click here for file
