# Supplementary figures and images for: Sequence variability of Rhizobiales orthologs and relationship with physico-chemical characteristics of proteins
Source: Biol Direct. 2011 Oct 4;6:48. doi: 10.1186/1745-6150-6-48 (PMC3198989; doi:10.1186/1745-6150-6-48)

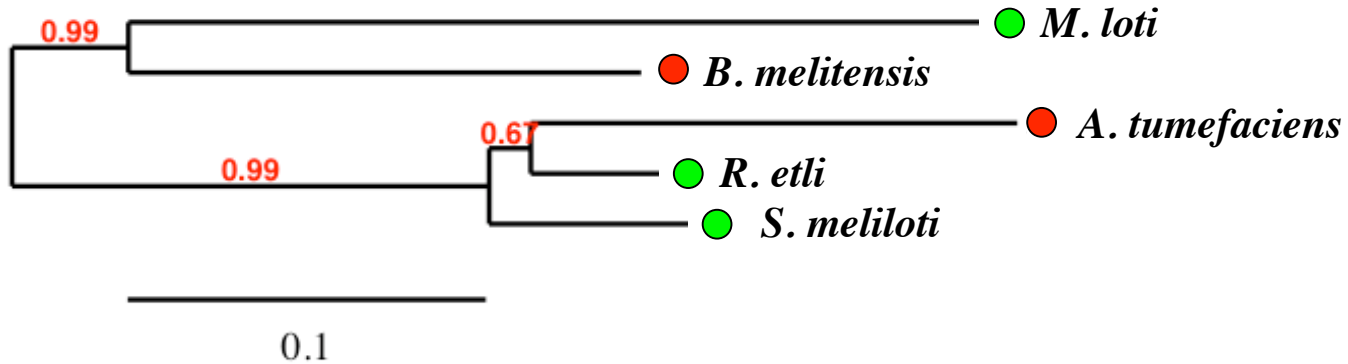

Supplement: Additonal file 1 — Phylogenetic tree of 16S rRNA genes. Color dots denote lifestyle: green, symbionts; red, pathogens. The tree was obtained with Phylip. Bar, substitutions per nucleotide. [file 1745-6150-6-48-S1.PDF]

Secondary structure

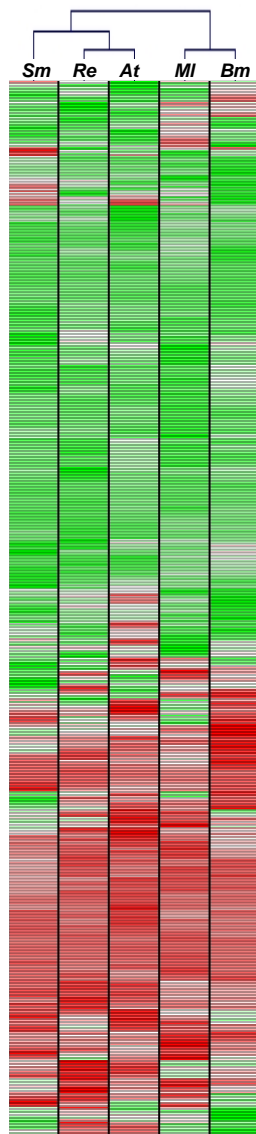

Volume

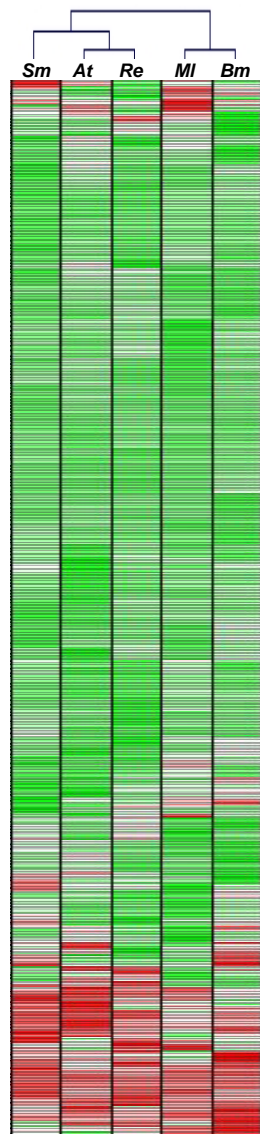

Electrostatic charge

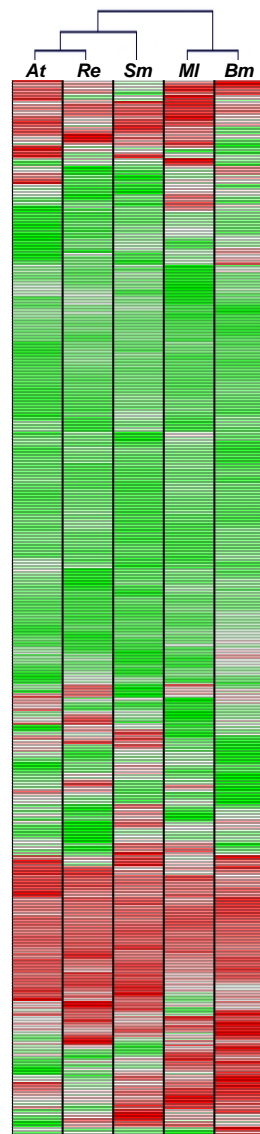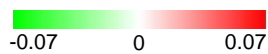

Supplement: Additional file 2 — Association of species by physico-chemical characteristics. Clustering was performed with MeV TM4 [23], with Pearson correlation. Each panel shows 705 syntenic ortholog products per each of the five species. Letters: At (A. tumefaciens), Re (R. etli CFN42), Sm (S. meliloti), Ml (M. loti), and Bm (B. melitensis). Scales for normalized values (green for negative values, white for zero, and red for positive). [file 1745-6150-6-48-S2.PDF]

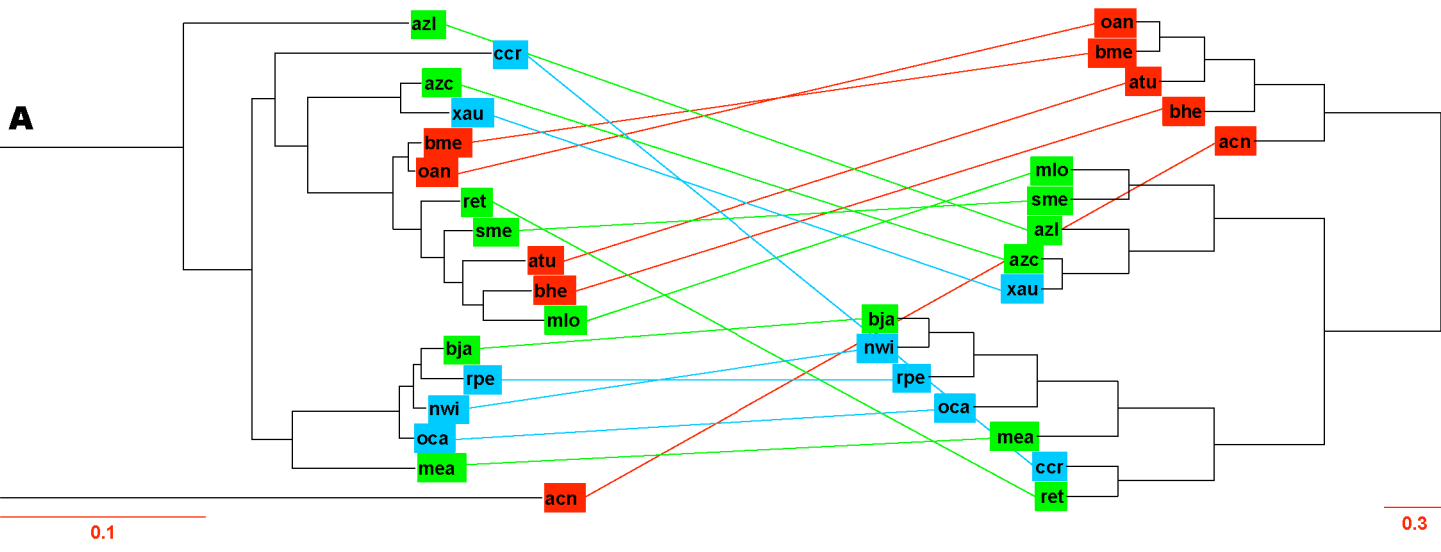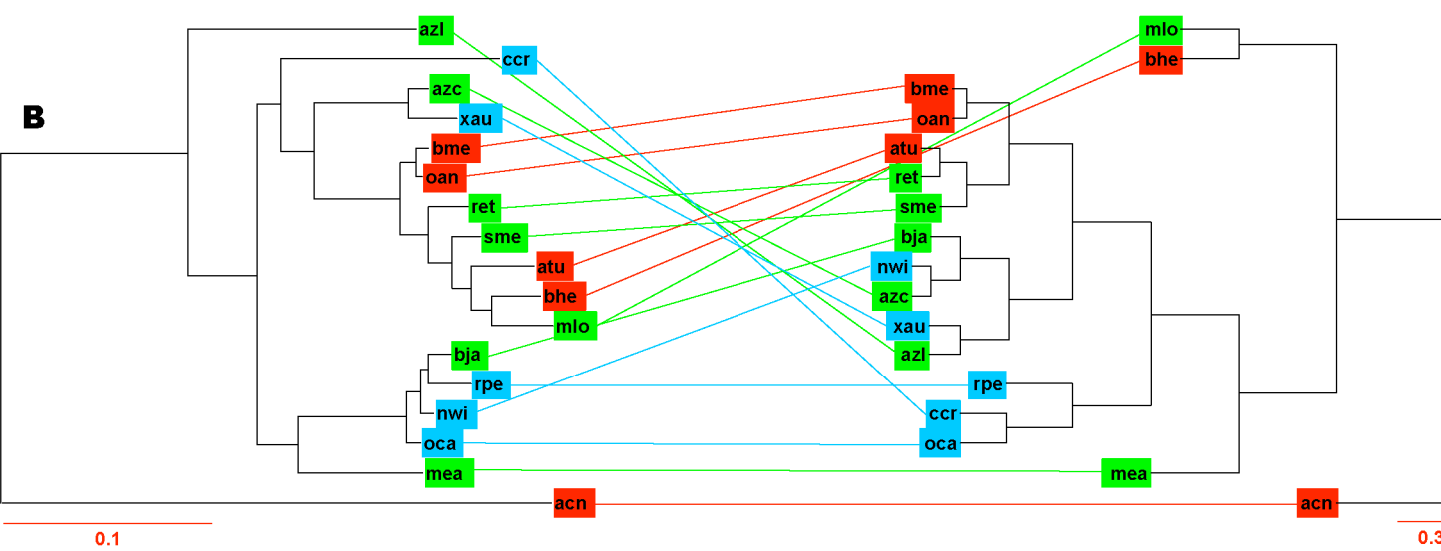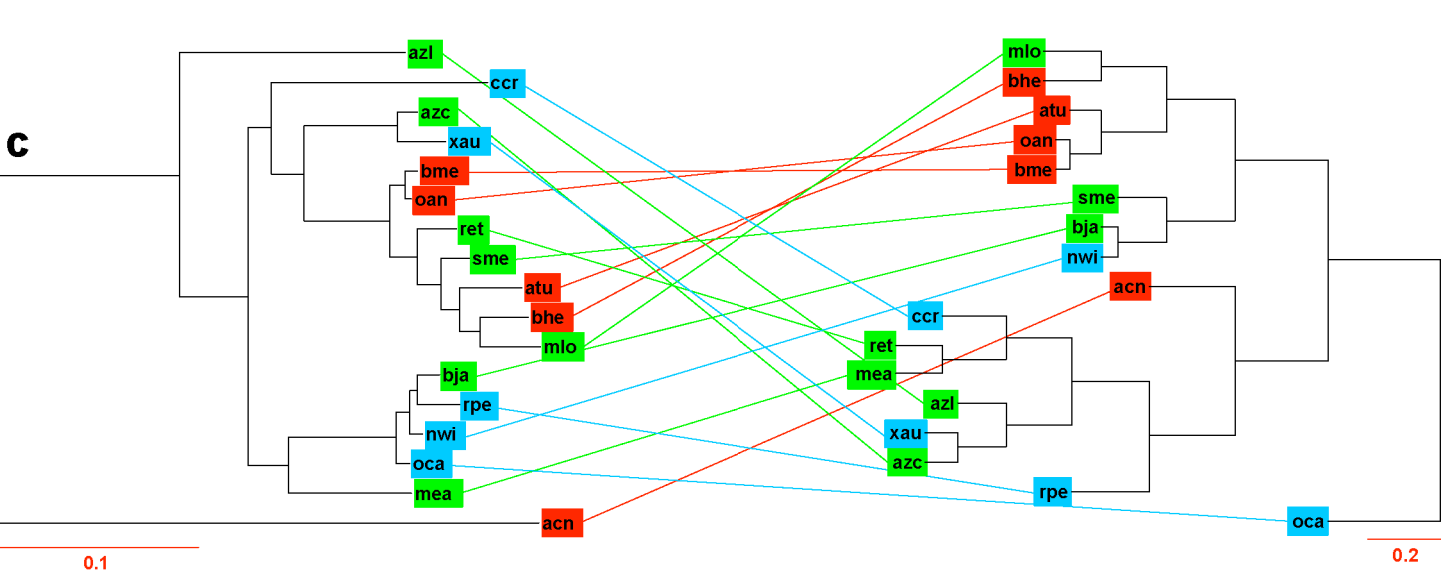

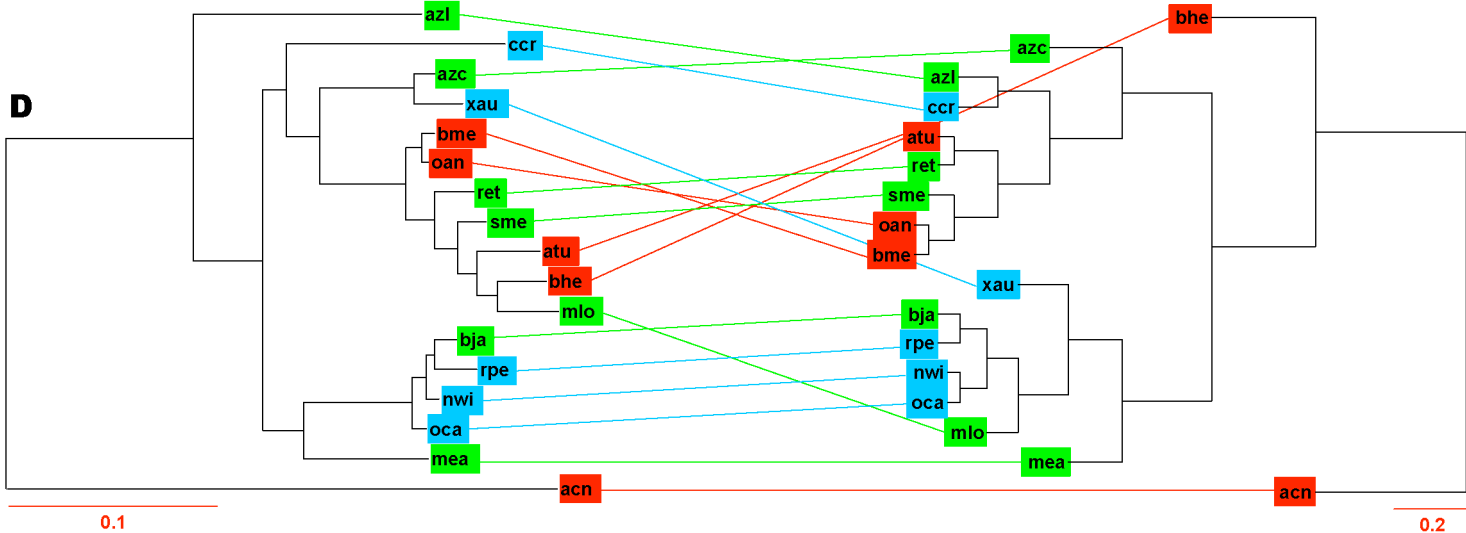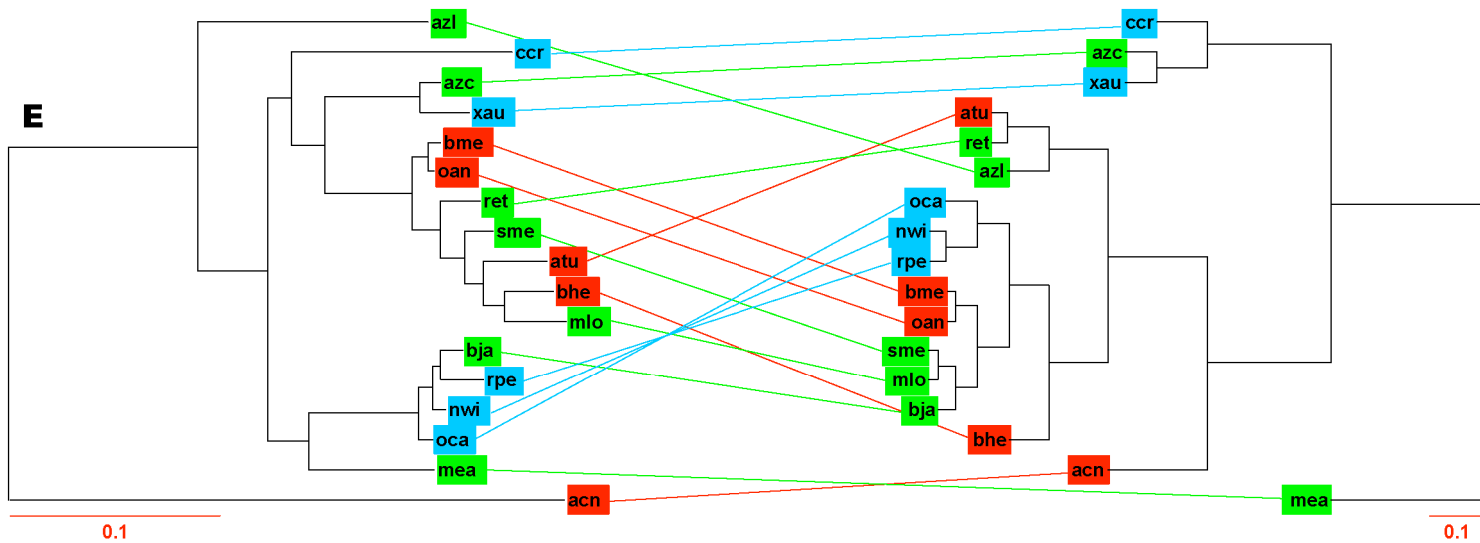

Supplement: Additional file 3 — Association of alpha-proteobacterial species by physico-chemical characteristics. Clustering was performed with MeV TM4 [23], hierarchical Pearson correlation, using 23 protein sequences and 17 species. Square color in name denotes lifestyle: green, plant-associated and symbiotic species; blue, free-living organisms; red, animal and plant pathogens. Left, 16S rRNA gene tree phylogeny, obtained with Phylip. Right, association of clusters from physico-chemical properties, as follows: A, amino acid composition; B, volume; C, electrostatic charge; D, secondary structure; and E, polarity. Protein sequences: ArgB, AtpA, AtpB, AtpC, AtpD, DnaJ, DnaK, FabD, FabH, NuoA, NuoB, NuoC, PurB, PurD, PurF, Tmk, AcnA, GltA, Mdh, Pgk, SdhA, SucA, and SucB. Organisms: Plant symbionts and plant-associated bacteria, additional to R. etli (ret), S. meliloti (sme), and M. loti (mlo): Bradyrhizobium japonicum UT26S (designated as bja, GenBank accession no. NC_014013.1), Azorhizobium caulinodans ORS571 (azc, NC_009937.1), Methylobacterium extorquens AM1 (mea, NC_012808.1), Azospirillum sp. B510 (azl, NC_013854.1). Free living species: Xanthobacter autotrophicus Py2 (xau, NC_009720.1), Rhodopseudomonas palustris BisA53 (rpe, NC_008435.1), Oligotropha carboxidovorans OM5 (oca, NC_015684.1), Nitrobacter winogradsky Nb-255 (nwi, NC_007406.1), Caulobacter crescentus CB15 (ccr, NC_002696.2). Animal and plant pathogens, additional to A. tumefaciens (atu) and B. melitensis (bme): Ochrobactrum anthropi ATCC 49188 (oan, NC_009668.1), Bartonella henselae Houston-1 (bhe, NC_005956.1), and Anaplasma centrale Israel (acn, NC_013532.1). Bars denote substitutions per nucleotide (16S rRNA tree) and arbitrary distances (physico-chemical properties), respectively. [file 1745-6150-6-48-S3.PDF]

# Secondary structure

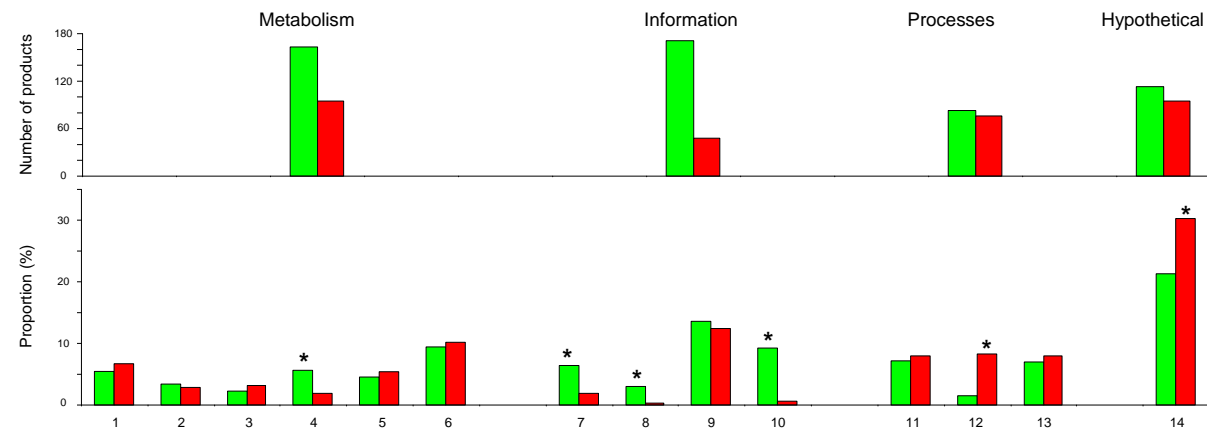

# Amino acid composition

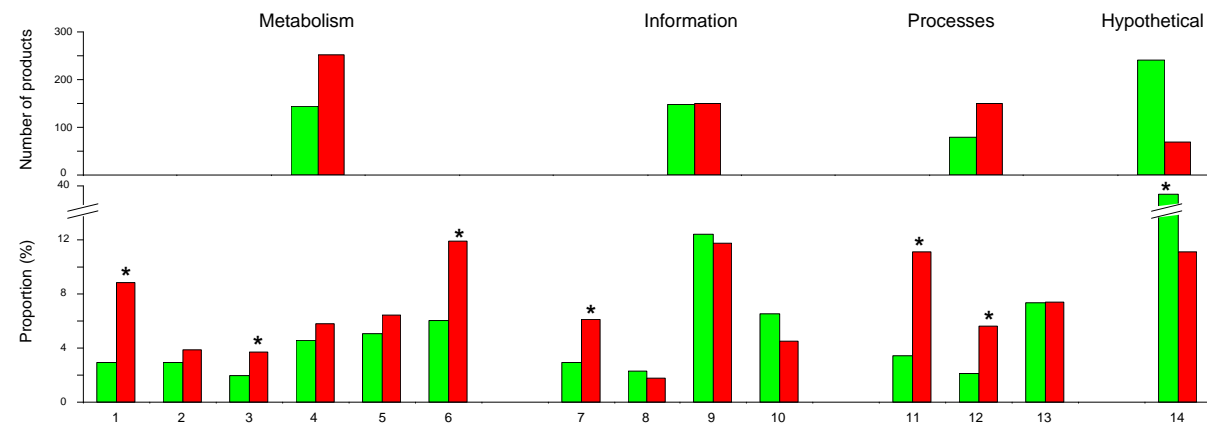

# Electrostatic charge

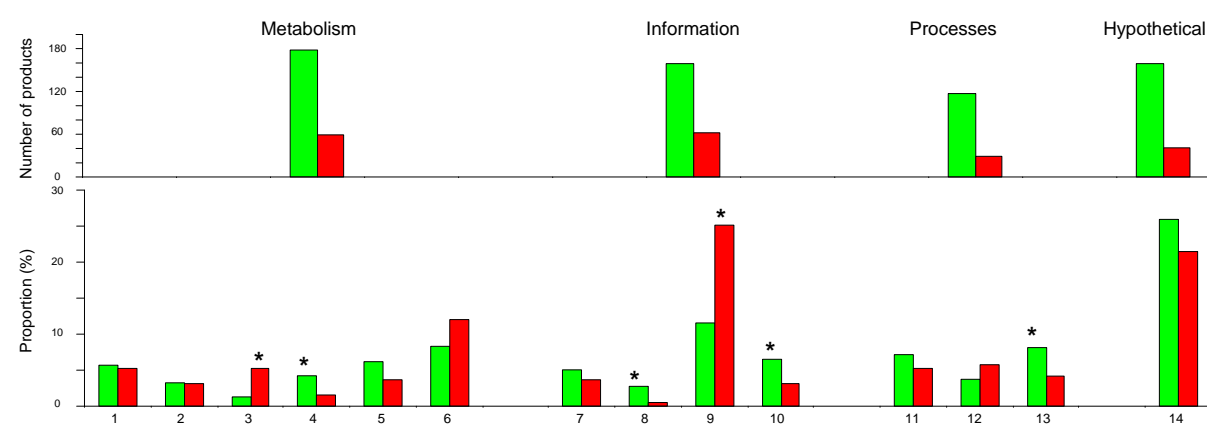

# Volume

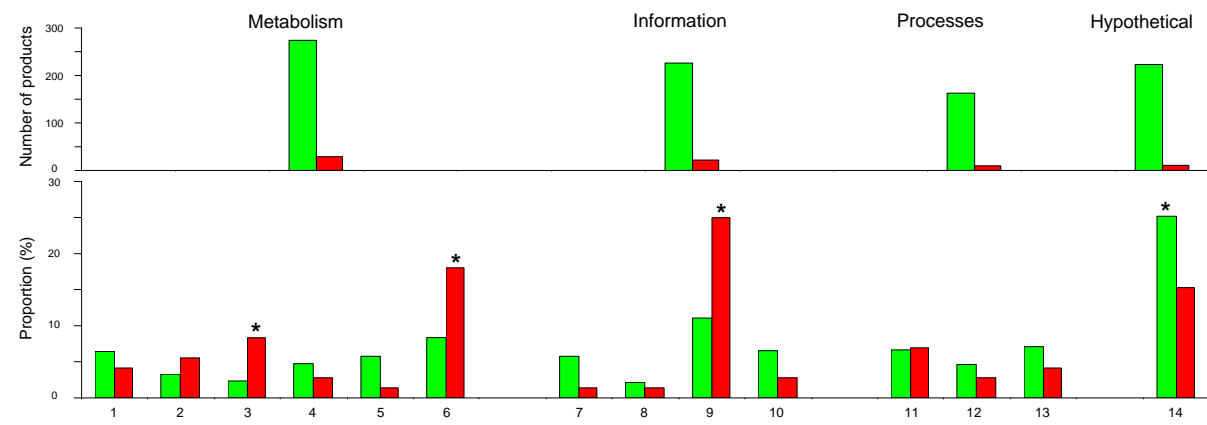

Supplement: Additional file 5 — Relationship between function and sign of values of ortholog products obtained with the series of values of physico-chemical properties. In the top row (from left to right), number of orthologs grouped into Metabolism (1, amino acid biosynthesis; 2, nucleotide biosynthesis; 3, fatty acid biosynthesis; 4, cofactor biosynthesis; 5, central intermediary metabolism; and 6, energy generation), Information (7, DNA metabolism; 8, transcription; 9, translation; and 10, transcriptional regulators), Processes (11, transport; 12, cellular envelope synthesis; and 13, cellular processes), and Hypothetical functions (14). In the bottom row, the functional classes in proportions. Asterisks denote significant differences with p < 0.05, Fisher's exact test. Bar colors: green, negative (low level for amino acid composition); red, positive (high level for amino acid composition). Scale for amino acid composition graph was broken to conserve the shape. [file 1745-6150-6-48-S5.PDF]

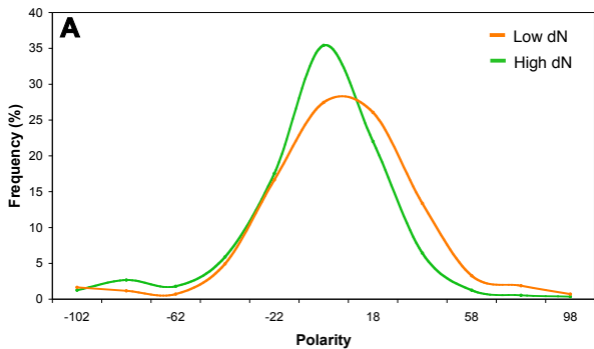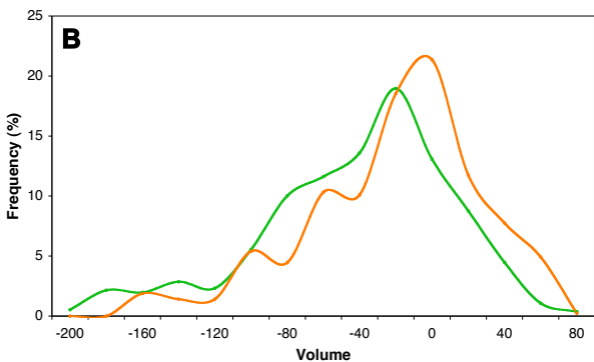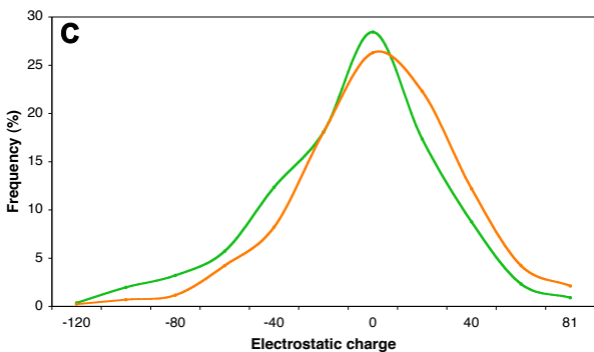

Supplement: Additional file 6 — Relationships between the nonsynonymous substitution rate (dN) and the physico-chemical properties. Frequency distribution curves of the orthologs divided in groups with low or high dN values. A, polarity. B, volume. C, electrostatic charge. Data from S. meliloti products. Low dN group, orange line. High dN group, green line. [file 1745-6150-6-48-S6.PDF]
