# Supplementary material for: Sequence variability of Rhizobiales orthologs and relationship with physico-chemical characteristics of proteins
Source: Biol Direct. 2011 Oct 4;6:48. doi: 10.1186/1745-6150-6-48 (PMC3198989; doi:10.1186/1745-6150-6-48)
Supplement: Additional file 4 — Characteristics of selected groups of products from clustering of polarity. [file 1745-6150-6-48-S4.DOC]

**Additional file 4.** **Characteristics of selected groups of products from clustering of polarity (Fig. 4A).**

| **Cluster** | Function | Most abundant sign in cluster | **% of changes by species, *R. etli* (average)** |
| --- | --- | --- | --- |
| 1 | Amino acid synthesis, Energy generation | - | 10.5 |
| 2 |  | +/- | 10.4 |
| 3 |  | - | 9.9 |
| 4 | Fatty acid synthesis, Transport | - | 9.6 |
| 5 | Nucleotides synthesis, Fatty acid synthesis, Central intermediary, Energy generation | - | 8.4 |
| 6 |  | - | 8.8 |
| 7 | Amino acid synthesis | - | 11.1 |
| 8 | Hypothetical | +/- | 11.3 |
| 9 | Transcription, Translation | + | 6.5 |
| 10 | Transcription, Translation | + | 7.8 |

*Only specific functions with significant abundance are shown (Fisher exact test, p<0.05).
