# Supplementary material for: Sequence variability of Rhizobiales orthologs and relationship with physico-chemical characteristics of proteins
Source: Biol Direct. 2011 Oct 4;6:48. doi: 10.1186/1745-6150-6-48 (PMC3198989; doi:10.1186/1745-6150-6-48)
Supplement: Additional file 7 — Values of physico-chemical properties for ArgC from Rhizobiales. [file 1745-6150-6-48-S7.DOC]

Additional file 7. Values of physiochemical properties for ArgC from Rhizobiales.

|  | **Physiochemical properties** | | | |
| --- | --- | --- | --- | --- |
| **Species** | **Polarity** | **Secondary structure** | **Volume** | **Electrostatic**  **charge** |
| ***S. meliloti*** | -19.901 | 3.676 | -66.051 | -19.997 |
| ***R. etli*** | -29.572 | 25.566 | -66.586 | -33.437 |
| ***A. tumefaciens*** | -16.413 | 14.031 | -40.026 | -7.358 |
| ***M. loti*** | -19.459 | -5.551 | -26.464 | -9.248 |
